# Reduced A-to-I editing of endogenous Alu RNAs in lung after SARS-CoV-2 infection^1^

**DOI:** 10.1016/j.crimmu.2021.04.001

**Published:** 2021-04-30

**Authors:** Philip S. Crooke, John T. Tossberg, Krislyn P. Porter, Thomas M. Aune

**Affiliations:** a Department of Mathematics, Vanderbilt University, Nashville, TN, 37212, USA; b Department of Medicine, Vanderbilt University Medical Center, Nashville, TN, 37212, USA; c Department of Pathology, Microbiology and Immunology, Vanderbilt University Medical Center, Nashville, TN, 37212, USA

**Keywords:** SARS-CoV-2, A-to-I editing, Alu RNAs, Double-stranded RNA sensors, A-to-I, adenosine-to-inosine, EEL, editing enriched location, IRs, inverted repeats, ISGs, interferon-stimulated genes, NHBE, normal human bronchial epithelial cells, RNA-seq, whole genome RNA-sequencing, SARS-CoV-2, severe acute respiratory syndrome coronavirus 2

## Abstract

Due to potential severity of disease caused by SARS-CoV-2 infection, it is critical to understand both mechanisms of viral pathogenesis as well as diversity of host responses to infection. Reduced A-to-I editing of endogenous double-stranded RNAs (dsRNAs), as a result of inactivating mutations in *ADAR*, produces one form of Aicardi-Goutières Syndrome, with an immune response similar to an anti-viral response. By analyzing whole genome RNA sequencing data, we find reduced levels of A-to-I editing of endogenous Alu RNAs in normal human lung cells after infection by SARS-CoV-2 as well as in lung biopsies from patients with SARS-CoV-2 infections. Unedited Alu RNAs, as seen after infection, induce IRF and NF-kB transcriptional responses and downstream target genes, while edited Alu RNAs as seen in the absence of infection, fail to activate these transcriptional responses. Thus, decreased A-to-I editing may represent an important host response to SARS-CoV-2 infection.

## Highlights

• SARS-CoV-2 infection of lung causes reduced A-to-I editing of Alu dsRNAs.
• Unedited Alu RNAs from infected cells activate IRF and NF-kB inducing downstream genes; edited Alu RNAs are inactive.
• Decreased A-to-I editing represents an important host response to SARS-CoV-2 infection.

## Graphical abstract

**Figure:**
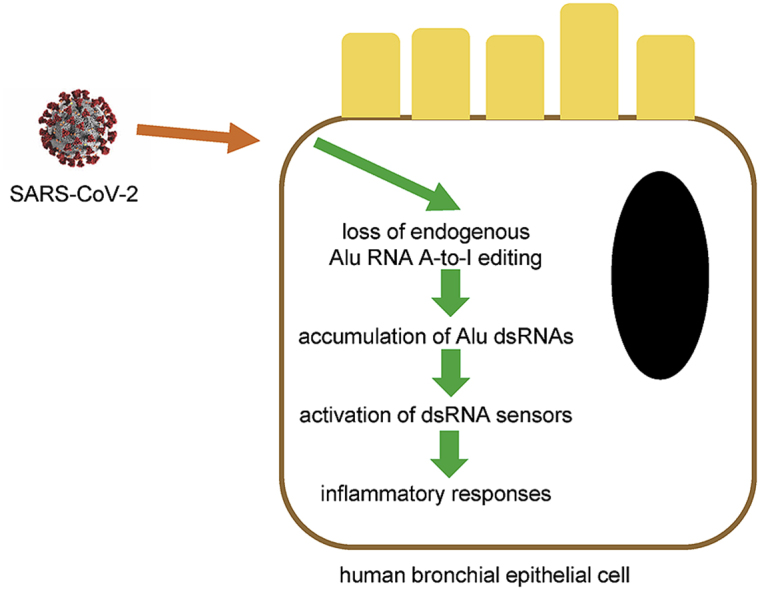

## 1 Introduction

Severe acute respiratory syndrome coronavirus 2 (SARS-CoV-2) infection also referred to as COVID-19 disease is a pandemic. Most infected individuals develop mild symptoms and spontaneously recover without complications. However, SARS-CoV-2 infection can result in severe respiratory illness, hospitalization, and even death, with a mortality rate of about 1% (Banerjee et al., 2020; Chen et al., 2020; Velavan and Meyer, 2020). Severe COVID-19 disease has been linked to a ‘cytokine storm’. This ‘cytokine storm’ or extreme inflammatory response is believed to be caused by marked increases in production of cytokines, including type 1/3 IFNs and interferon-stimulated genes (ISGs), IL-6, TNF-α, chemokines, and other pro-inflammatory mediators. This hyperactive immune response in response to SARS-CoV-2 infection is directly correlated with unfavorable outcomes for these patients (Channappanavar and Perlman, 2017; Ragab et al., 2020).

Coronaviruses are single-stranded RNA viruses (Fehr and Perlman, 2015; V'kovski et al., 2020). In general terms, pattern recognition receptors are employed by the immune system as an innate mechanism to recognize and respond to conserved molecular structures expressed by pathogens. For example, viral RNAs are recognized by pattern recognition receptors TLR3 and the DExD/H-box helicases, RIG-I and MDA5 (Ahmad et al., 2018; Lassig and Hopfner, 2017). Activation of these pattern recognition receptors results in stimulation of transcription factors, NF-kB and IRFs, leading to induction of genes encoding IFNs and ISGs to inhibit viral replication and additional cytokines, chemokines and immune mediators to further activate host immune responses (Portal et al., 2015; Schoggins et al., 2011).

Similar immune responses are also observed in the absence of viral infection. For example, Aicardi-Goutières Syndrome is a lethal pro-inflammatory human disorder. One form of Aicardi-Goutières Syndrome is caused by an inactivating mutation in *ADAR* (Rice et al., 2012), the gene that encodes adenosine deaminase specific for double-stranded RNA (dsRNA), ADAR, responsible for deaminating adenosines in dsRNAs to inosine, termed A-to-I editing (Eisenberg and Levanon, 2018; Lamers et al., 2019; Liddicoat et al., 2015; Pestal et al., 2015). In Aicardi-Goutières Syndrome, loss of ADAR function results in accumulation of endogenous dsRNAs, activation of dsRNA sensors, and induction of IFNI/III, ISGs, additional inflammatory mediators and death in both murine models and human syndromes (Chen and Yang, 2017; Eisenberg and Levanon, 2018; Pfaller et al., 2018; Samuel, 2019; Yang et al., 2014). Increased levels of IFNs, ISGs and increased inflammatory immune responses are also seen in certain autoimmune diseases (Baechler et al., 2003; Lubbers et al., 2013; Nezos et al., 2015). In multiple sclerosis, loss of A-to-I editing of endogenous RNAs is also found and may contribute to elevated immune responses observed in these patients (Heinrich et al., 2019; Tossberg et al., 2020b).

Alu elements are short interspersed nuclear DNA elements (SINE) of about 300 bp, originated from a head-to-tail fusion of two 7S RNA moieties, are unique to primates, and make up about 10% of the human genome (Deininger, 2011). Alu elements are transcribed by RNA polymerase II as pre-mRNAs and by RNA polymerase III as part of their normal life cycle and may form dsRNAs because of their repetitive structure (Chen and Yang, 2017). In humans, ADAR catalyzed A-to-I editing predominantly occurs within Alu RNA elements. Intramolecular pairs of Alu elements oriented in opposite directions in close proximity in the genome, also referred to as inverted repeats (IRs), are a major target for A-to-I editing (Athanasiadis, 2004). The general view is that A-to-I editing causes dsRNA to lose its complementarity due to I being read as a G so the A's can no longer base pair with T's to form double-stranded structures. As such, Alu RNAs, either as monomers or as IRs, if unedited may trigger dsRNA sensors and activate IFN- and NF-kB-responses. A-to-I editing of these dsRNA structures is thought to prevent recognition by dsRNA sensors (Liddicoat et al., 2015). Thus, A-to-I editing of Alu RNA is a critical ADAR function and the balance of editing is a physiological process regulating levels of IFN I/III affecting numerous biological systems. For example, transient loss of ADAR and concomitant loss of A-to-I editing is necessary for efficient antiviral activity in response to certain viral infections (Li et al., 2016). Thus, decreased A-to-I editing is beneficial to the host in certain circumstances but greater reduction in A-to-I editing can produce detrimental outcomes (Eisenberg and Levanon, 2018; Lamers et al., 2019).

Our studies show that infection of normal human bronchial epithelial cells (NHBE) with SARS-CoV-2 results in reduced A-to-I editing of endogenous Alu RNAs. Alu RNAs unedited in response to SARS-CoV-2 infection are potent activators of downstream IRF- and NF-kB mediated transcriptional responses and induce expression of downstream target genes. Edited Alu RNAs, as detected in uninfected NHBE, do not activate these transcriptional responses and induce similar downstream target genes. We also observe reduced levels of A-to-I editing in lung biopsies from SARS-CoV-2 infected individuals compared to uninfected individuals. Thus, loss of A-to-I editing of endogenous Alu RNAs may contribute to altered immune responses and the ‘cytokine storm’ observed in these severely ill patients.

## 2 Materials and methods

### 2.1 A-to-I editing

We employed whole-genome RNA-sequencing (RNA-seq) files from NCBI Gene Expression Omnibus for analysis of A-to-I editing (GSE 147507). These files included six replicates of NHBE cells (5 ​× ​10^5^/culture) infected with SARS-CoV-2 (SARS-CoV-2 isolate USA-WA1/2020 (NR-52281)) at a multiplicity of infection of 2 for 24 ​h (Blanco-Melo et al., 2020). We also employed RNA-seq files form uninfected human lung biopsies (N ​= ​4) and human lung biopsies from patients infected with SARS-CoV-2 obtained post-mortem (N ​= ​4) that were used as biological replicates, also contained in GSE 147507, all derived from males older than 60 years; see (Blanco-Melo et al., 2020) for details. As a quality control step, we determined the sum of total read counts of all mRNAs from RNA-seq files. We found that each replicate from the mock and SARS-CoV-2 infected NHBE cell cultures had similar total read counts that were not statistically different (not shown).

We employed the following workflow we have previously developed to determine A-to-I editing sites in endogenous host RNAs from paired FASTQ sequencing files (Tossberg et al., 2020a). A python-based package called the SPRINT toolkit was the main identification tool (Zhang et al., 2017). This multithread toolkit accepted sequence files and produced text files with the following information for each edit site: (1) genomic location of editing site; (2) type of edit (e.g., A-to-G; T-to-C); (3) strand (“+” or “-“); (4) number of edits per site and (5) total number of reads per site. *Mathematica* programs were developed to synthesize data: numbers of samples in groups with unique editing sites, that shared editing sites, mean numbers of total reads and edits for each editing site, and editing sites common and unique to group pairs (e.g., HC versus COV). This information was tied to an Alu database to annotate each site: genes, genomic locations (exons, introns, ncRNA, intergenic, 3’ UTR), and if sites were Alu or non-Alu elements (Dagan et al., 2004). To create genome-wide A-to-I editing indices, we identified all A-to-I editing sites present in one sample and summed edit/read ratios for all editing sites across the entire genome for each sample (Tossberg et al., 2020a).

### 2.2 Synthesis and testing of Alu RNAs

Alu DNA sequences were obtained from the GrCh37 (hg19) assembly. A SP6 promoter was added to the 5′ end and synthetic dsDNAs were obtained from Integrated DNA Technologies (Tossberg et al., 2020a). RNA transcription was performed using dsDNA templates and Megascript SP6 (Invitrogen) in overnight reactions at 37 ​°C. Reaction products were treated with Turbo DNase, precipitated with lithium chloride and purified using an RNeasy MiniElute Cleanup Kit (Qiagen). Absorbance was determined at 260 ​nm to quantitate yields. Agarose gel electrophoresis was performed to ensure that the single-stranded Alu RNAs were of the predicted size. Alu RNAs were not treated with phosphatases to remove 5’ phosphate groups. We designed unedited Alu DNA templates. We also changed A nucleotides that were edited in mock-infected cells but not SARS-CoV-2 infected cells to G nucleotides as a mimic of A-to-I editing.

THP-1 reporter cell lines (Invivogen) contained stably integrated luciferase genes under the control of either an IFN-stimulated response element (ISRE) or NF-kB response element. Cells were plated at 10,000 ​cells/100μl/well in 96-well plates in RPMI-1640 media with 10% fetal bovine serum, penn/strep and L-glutamine and cultured in a humidified incubator with 5%CO_2_ in air at 37 ​°C. Transfections were performed using Lipofectamine® RNAiMAX (ThermoFisher Scientific) essentially as previously described (Gibbons et al., 2018). Luciferase activity was determined 24 ​h after transfection using luciferin substrate (Invivogen) and light emission measured with a TD20/20 luminometer. Gene expression measurements were determined by quantitative PCR performed essentially as previously described (Heinrich et al., 2019; Tossberg et al., 2020b).

## 3 Results

### 3.1 Reduced A-to-I editing in SARS-Cov-2 infected NHBE

**Fig. 1:**
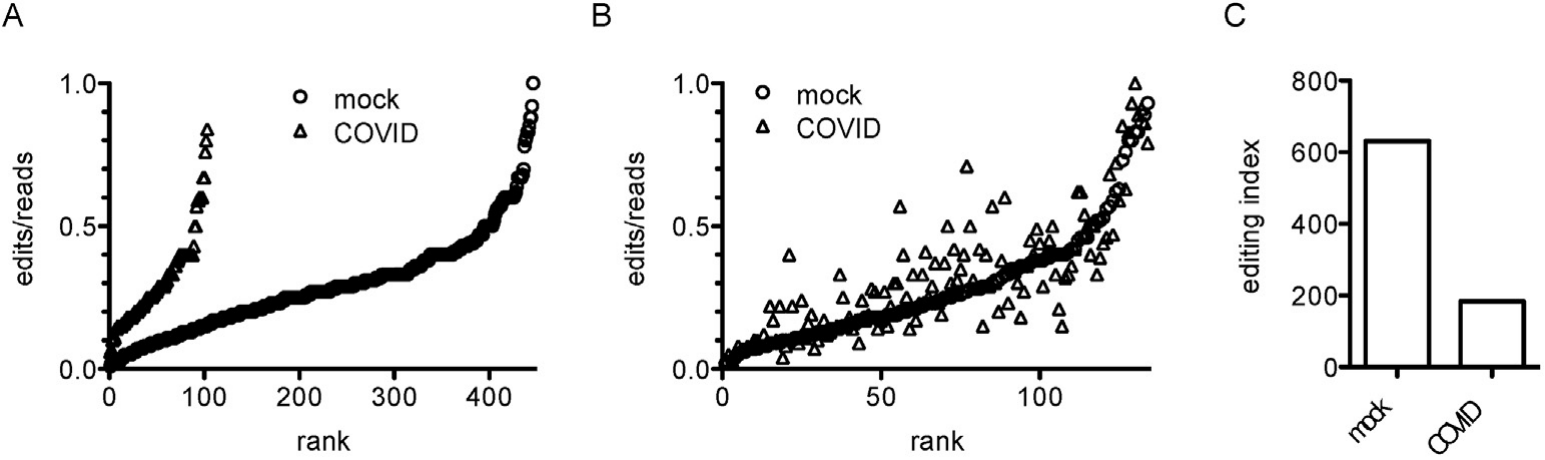
Reduced A-to-I editing in NHBE infected with SARS-CoV-2. A) Number of unique A-to-I editing sites present in all mock-infected replicates (open circles), or all SARS-CoV-2 infected NHBE replicates (open triangles), X-axis is rank from lowest to highest proportions of edits to reads, Y-axis is average proportion of edits to reads at each editing site, P ​< ​0.0001, χ^2^ analysis. Variance of edit/read ratios at each editing site was <10% among replicates so error bars are not shown. B) Number of unique sites that are A-to-I edited in all mock-infected and all SARS-CoV-2 infected NHBE cultures ranked according to mock-infected edit/read ratios (open circles); corresponding SARS-CoV-2 edit/read ratios at identical nucleotide sites are shown (open triangles), P ​> ​0.05, χ^2^ analysis. Variance of edit/read ratios at each editing site was <10% among replicates so error bars are not shown. C) Genome-wide A-to-I editing index mock-infected and SARS-CoV-2 infected cultures. Editing index is the sum of all proportions of edits/reads in all replicate samples, P ​< ​0.0001, unpaired *t*-test with Welch's correction.

We examined RNA-seq data from NHBE mock-infected or infected with SARS-CoV-2 (multiplicity of infection (MOI) ​= ​2) using the SPRINT software package to identify differences in A-to-I editing of endogenous RNAs. We used two criteria to define A-to-I editing sites for these analyses. First, we required the fraction of A-to-I edits to exceed 5% of total reads at individual nucleotide sites and second, we required total read counts >5 to guard against possible sequencing errors. To examine the more common editing sites, we identified editing sites present in 100% of mock-infected NHBE replicates, present in 100% of SARS-CoV-2 infected NHBE replicates, or present in 100% of mock-infected and 100% of SARS-CoV-2 infected NHBE replicates (Fig. 1A and B).

We identified >400 common A-to-I editing sites present in all mock-infected NHBE replicates but absent from all SARS-CoV-2 infected NHBE replicates (Fig. 1A). Average proportion of edits to reads ranged between 0.05 and 1.0. In contrast, we identified <100 common A-to-I editing sites present in SARS-CoV-2 infected NHBE replicates but absent from mock infected NHBE replicates. Average proportion of edits to reads in SARS-CoV-2 infected NHBE replicates was also reduced, ranging from only 0.05–0.80. We identified about 125 common A-to-I editing sites shared between all mock infected and all SARS-CoV-2 infected NHBE replicates (Fig. 1B). Average proportion of edits/reads at these shared editing sites was not statistically different between mock infected and SARS-CoV-2 infected replicates. We also created an A-to-I editing index by multiplying proportions of edits to reads at each editing site by the number of replicate samples with edits at these sites and summed these values across the entire genome. This genome-wide A-to-I editing index was also reduced in SARS-CoV-2 infected NHBE compared to mock infected NHBE (Fig. 1C). We conclude from these data that genome-wide A-to-I editing of endogenous RNAs was reduced in SARS-CoV-2 infected NHBE compared to mock infected NHBE. Further, editing at individual common sites can be subdivided into three categories; 1) those that were edited in mock infected NHBE and editing was lost in response to SARS-CoV-2 infection, 2) those where editing was unaffected by SARS-CoV-2 infection and 3) a small number of sites in which editing was induced by SARS-CoV-2 infection. We found a similar pattern in SARS-CoV-2 infected dendritic cells (Crooke, 2021).

### 3.2 Gene-specific differences in A-to-I editing

**Fig. 2:**
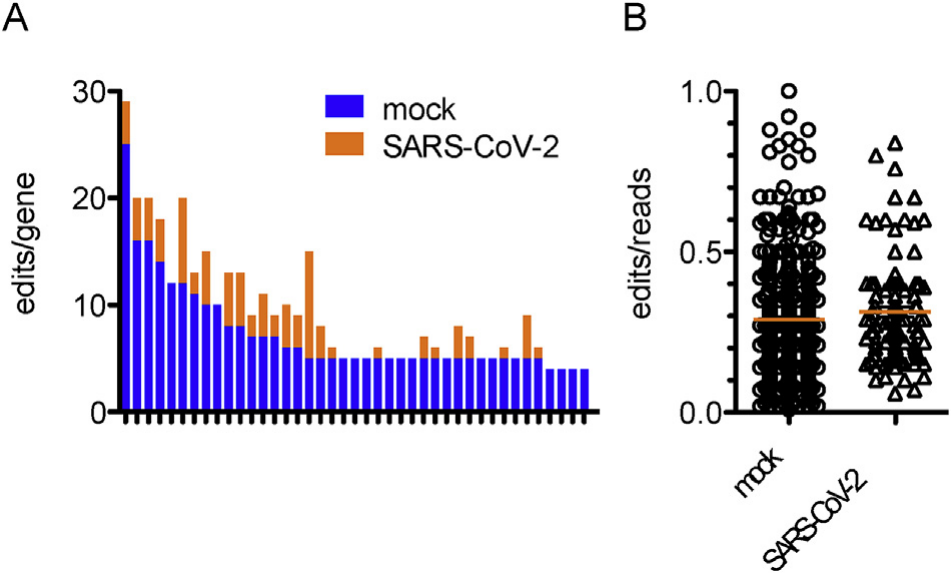
SARS-CoV-2 infection of NHBE reduces A-to-I editing sites per gene. A) Genes were identified with >5 A-to-I edit sites per gene in mock or SARS-CoV-2 infected NHBE. Results are expressed by employing a stack plot as total number of edit sites per gene in mock (blue spikes) or SARS-CoV-2 (orange spikes) infected NHBE, P ​< ​0.0001, two-way ANOVA; gene list: *CTSB, MAVS, PDDC1, UGGT1, ELF2, RPL37A, NDUFS1, LOC100505876, SNRPD3, AHR, F11R, PSMB2, CFLAR, POLH, PRR11, TLCD2, AP1S3, ATG14, CD46, ELOVL5, KLC1, MBD3, PHAX, RPP14, SYAP1, TXNDC15, PNPLA3, UPP1, ZNF337, ZNF587, CCDC125, CENPN, CSAD, DFFA, GNB4, LONP2, LYRM7, PHACTR4, RP4-785G19.5, TGOLN2, TMEM154.* B**)** Range of proportions of edits/reads at all editing sites detected in ≥1 mock infected or ≥1 SARS-CoV-2 infected replicate, orange line is average proportion of edits to reads, P ​> ​0.05, *t*-test with Welch's correction.

The majority (>95%) of all A-to-I editing sites we identified in NHBE were within intragenic regions, either introns or 3′UTRs. We compared the total number of A-to-I editing sites per gene between mock-infected and SARS-CoV-2 infected NHBE. We identified a total of 507 unique genic editing sites in mock-infected NHBE but only 155 unique genic editing sites in SARS-CoV-2 infected NHBE. To further illustrate these differences, we identified those genes with ≥5 edited sites per gene in mock-infected and SARS-CoV-2 infected NHBE and ranked them according to total number of edited sites per gene. Overall, when all genes with ≥5 editing sites were compared, number of edited sites per gene was significantly greater in control NHBE than infected NHBE (Fig. 2A). Of the nucleotide sites that were edited in control or infected NHBE, overall proportion of edits to reads was not statistically different (Fig. 2B). Overall, we interpret these results to indicate that certain A-to-I editing sites that were edited in control NHBE were not edited in SARS-CoV-2 infected NHBE.

### 3.3 Genome-wide editing-enriched locations (EELs)

**Fig. 3:**
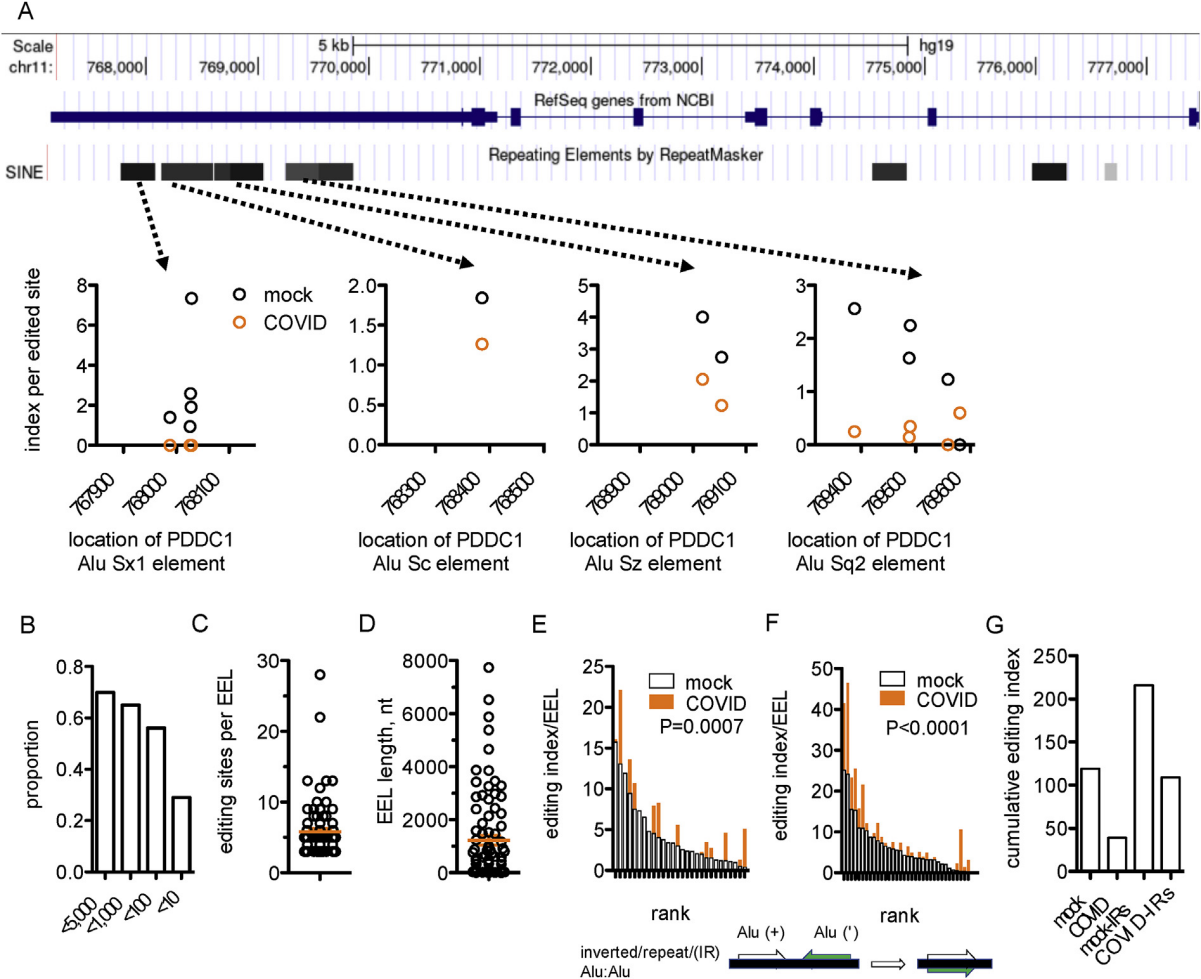
A) Loss of A-to-I editing at EELs after NHBE SARS-CoV-2 infection. A) Human *PDDC1* genomic location, upper tracks, introns, exons, 5′UTR, 3′UTR, middle tracks, positions of SINE (Alu) elements, lower tracks. Dashed arrows identify four A-to-I edited Alu elements that make up the PDDC1 EEL. Graphs connected by the arrows show levels of A-to-I editing at these Alu RNAs. Y-axis is the A-to-I editing index at each edited site determined by calculating the proportion of edits/reads multiplied by the number of edited replicate samples. B) Genomic distances between NHBE Alu RNA A-to-I editing sites. Y-axis is the proportion of all A-to-I editing sites within the indicated distance from an adjacent Alu A-to-I editing site, X-axis is the genomic distance between one A-to-I editing site and the nearest A-to-I editing site. C) Number of A-to-I editing sites per EEL determined by performing genome-wide scans and counting editing sites within a moving window of 5000 nucleotides. Orange line is mean editing sites per EEL. D) Distribution of EEL lengths in nucleotides across the human genome. Orange line is mean EEL length. E) Differences in A-to-I editing indices per EEL without Alu IRs in mock and SARS-CoV-2 infected NHBE. Stack plot ranks editing indices of individual EELs in mock-infected cultures from highest to lowest, open spikes. Orange spikes are corresponding EEL editing indices in SARS-CoV-2 infected NHBE, P ​< ​0.0001, two-way ANOVA). F) Differences in A-to-I editing indices per EEL containing Alu:Alu IRs in mock and SARS-CoV-2 infected NHBE. Stack plot ranks editing indices of individual EELs in mock-infected cultures from highest to lowest, open spikes. Orange spikes are corresponding EEL editing indices in SARS-CoV-2 infected NHBE, P ​< ​0.0001, two-way ANOVA. Cartoon below E & F illustrates Alu:Alu IR. G) Genome-wide cumulative editing indices in EELs without Alu IRs from mock and SARS-CoV-2 infected NHBE (left columns) or with Alu IRs (right columns).

Closer inspection revealed that >95% of NHBE genome-wide A-to-I editing sites were located within Alu elements present in introns and 3′UTRs of either protein-coding genes or long non-coding RNA genes. The *PDDC1* gene served as an example (Fig. 3A). A total of 13 unique A-to-I editing sites were detected and all were located within the 3′UTR spanning a distance of about 2000 bp. Within this 2000 bp EEL, these 13 edited sites were located within four of six Alu elements in the 3′ UTR. Of these, individual Alu elements possessed 5, 1, 2, and 5 editing sites, respectively (arrows identify Alu targets of A-to-I editing and graphs below each arrow show editing index at each edited site in mock- and SARS-CoV-2 infected NHBE within the Alu RNA and these Alu RNAs displayed different editing indices in mock and SARS-CoV-2 -infected NHBE. Two of four Alu elements were edited equivalently in mock and SARS-CoV-2 -infected NHBE while the other two Alu elements within the PDDC1 3′ UTR were only edited in mock-infected NHBE. Taken together, these results raise the possibility that SARS-CoV-2 infection of NHBE disrupted A-to-I editing of certain Alu RNAs but not other Alu RNAs and that A-to-I editing sites may not be randomly distributed across the genome but may be preferentially found in EELs.

To explore these notions further, we identified all A-to-I editing sites detected in both mock and SARS-CoV-2 infected NHBE and determined distances between editing sites. We found that most A-to-I editing sites were clustered near other A-to-I editing sites with >60% of all A-to-I editing sites within 1000 bp of an adjacent editing site and >50% of all A-to-I editing sites within 100 bp of an adjacent A-to-I editing site (Fig. 3B). To define EELs, we scanned the genome using a 5000 bp moving window and required that EELs contain 3 or greater A-to-I editing sites within this 5000 bp window. Using these criteria, we found that >70% of all A-to-I editing sites were localized within EELs containing on average about 6 editing sites per EEL (Fig. 3C). Average EEL length was about 1200 bp (Fig. 3D). These analyses confirmed that NHBE genome-wide A-to-I editing sites were confined to relatively small regions or EELs.

Next, we compared A-to-I editing within EELs between SARS-CoV-2 and mock infected NHBE. To do so, we created an EEL editing index by summing proportions of edits/reads at each editing site within an EEL across all SARS-CoV-2 infected or mock infected NHBE replicates. We also sub-divided EELs into those that did not contain Alu IRs (Fig. 3E) and those that did contain Alu IRs (Fig. 3 F). The cartoon below Fig. 3 E, F illustrates an Alu:Alu IR and shows how IRs may hybridize to form a double stranded structure if the two Alu elements have sufficient complementarity. We found that editing indices of certain EELs were approximately equivalent in SARS-CoV-2 infected and mock infected NHBE replicates. However, the majority of EELs exhibited high editing indices in mock infected NHBE but very low to absent editing indices in SARS-CoV-2 infected NHBE and this was the case for EELs that did not (Fig. 3E) or did (Fig. 3F) contain Alu:Alu IRs. The genome-wide cumulative editing index of both classifications of EELs, without or with IRs, was reduced in SARS-CoV-2 infected NHBE compared to mock-infected NHBE (Fig. 3G). Thus, SARS-CoV-2 infection of NHBE resulted in loss of A-to-I editing at most EELs while editing at a small number of EELs was largely unaffected by SARS-CoV-2 infection.

### 3.4 Activation of IRF- and NF-kB-transcriptional responses by unedited and edited Alu RNAs

**Fig. 4:**
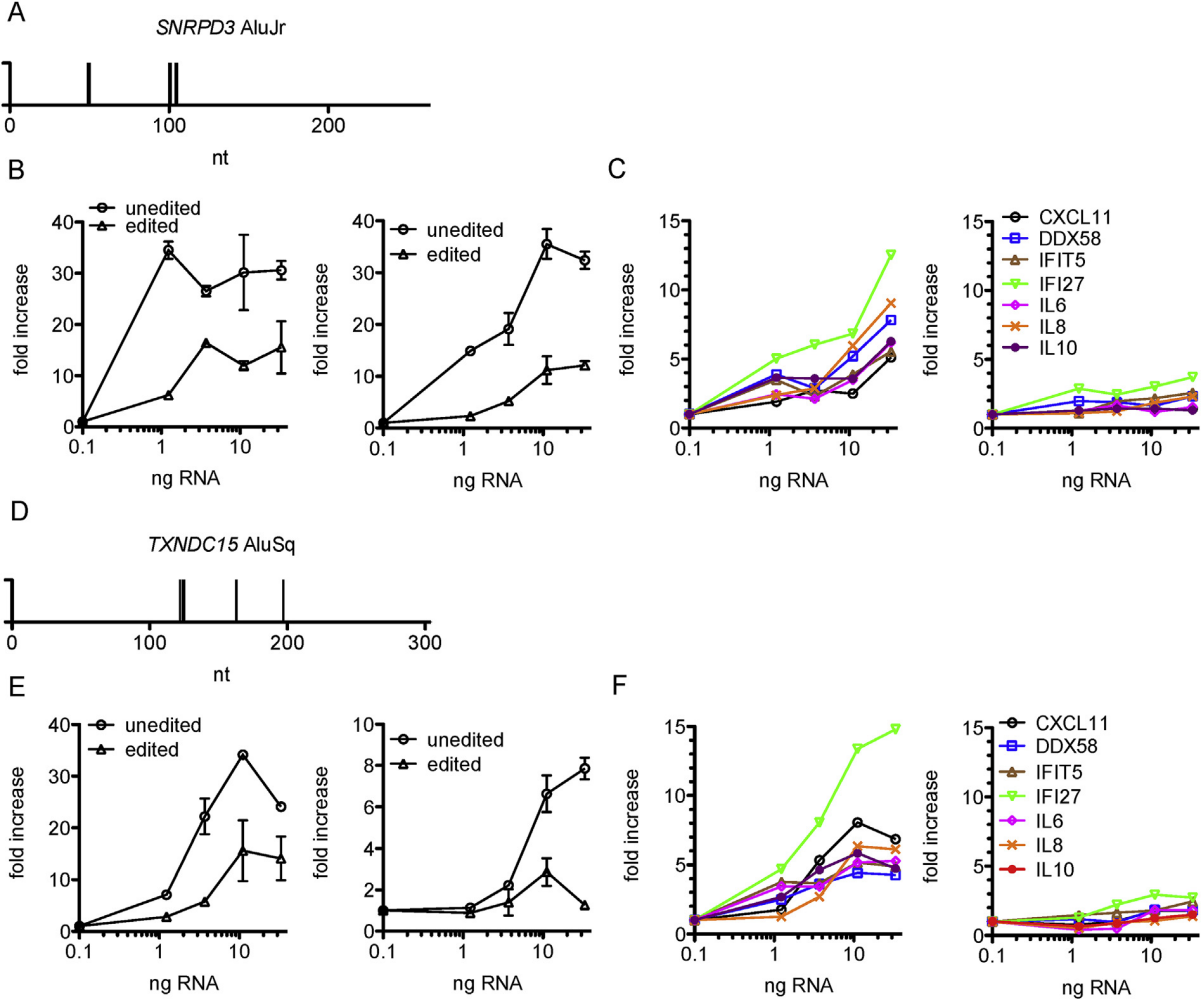
ISRE- and NF-kB activation and induction of target genes by unedited and edited Alu RNAs. A) Locations (spikes) of all A-to-I edited sites in mock infected NHBE but not SARS-CoV-2 infected NHBE in the SNRPD3 AluJr RNA. B) Activation of IRF (left graph) and NF-kB (right graph) responses by unedited and edited SNRPD3 AluJr. Fold increases were calculated as AluJr transfected response/mock transfected response in light units, average of three biological replicates, P ​< ​0.0001, two-way ANOVA. C) Induction of the indicated IRF and NF-kB response genes by unedited and edited SNRPD3. Fold increases were calculated as AluJr transfected response/mock transfected response of each gene determined by quantitative PCR after normalization to levels of *HPRT*, average of two biological replicates, P ​< ​0.05 for each gene, two-way ANOVA. D-F) As in A-C but activities of unedited and edited TXNDC15 AluSq RNA are shown, (E) Both IRF and NF-kB responses, P ​< ​0.0001, two-way ANOVA, (F) P ​< ​0.05 for each gene, two-way ANOVA.

Genomic Alu SINE elements are about 300 nucleotides in length. Within EELs, we identified highly expressed Alu RNAs that were unedited in SARS-CoV-2 infected NHBE but were edited in mock infected NHBE. Of these, only 5-7 adenosines were edited to inosine in the entire Alu RNA; the AluJr element within *SNRPD3* and the AluSq element within TXNDC15 are two representative examples (Fig. 4 A,D). To produce a mimic of A-to-I editing, we replaced edited A's with G's in the DNA template and these were in vitro transcribed into Alu RNAs. Unedited and edited Alu RNAs were tested for their ability to be recognized by dsRNA sensors and stimulate downstream transcriptional responses using THP-1 reporter cells with a stably integrated luciferase gene under the control of either an IRF response element or a NF-kB response element. We found that unedited Alu RNAs, as detected in SARS-CoV-2 infected NHBE, but not edited Alu RNAs, as detected in mock-infected NHBE, activated both IRF- and NF-kB-transcriptional responses (Fig. 4B,E, see legend). For comparison, peak stimulations of IRF- and NF-kB transcriptional responses by poly I:C required 10–30 ​ng RNA per culture and yielded only 5–10 fold increases in responses over baseline (Tossberg et al., 2020b). We also found that unedited Alu RNAs, but not edited Alu RNAs, induced expression of *IL6*, *IL8*, *IL10*, and ISGs, *DDX58*, *IFIT5*, *CXCL11* and *IFI27* (Fig. 4C,F). For comparison, peak gene induction by poly I:C required 10–30 ​ng RNA per culture and yielded only 3–5 fold increases in responses over baseline (Tossberg, 2020b). Thus, the small number of edits detected in mock-infected NHBE were sufficient to reduce ability of Alu RNAs to activate dsRNA sensors, downstream IRF and NF-kB transcriptional responses and induce target gene expression.

### 3.5 COVID-19 patient lung exhibits reduced A-to-I editing of Alu RNAs

**Fig. 5:**
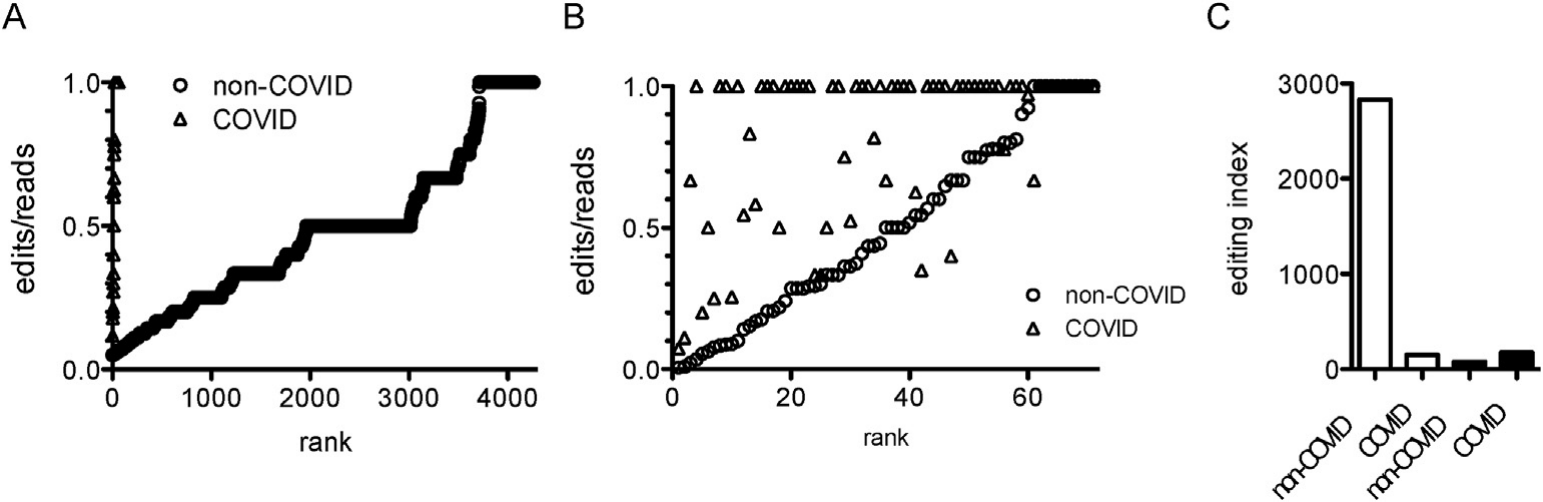
Reduced A-to-I editing in response to SARS-CoV-2 infection, *in vivo*. A) Number of unique A-to-I editing sites present in all non-COVID and absent from all COVID lung biopsies (open circles) or present in all COVID lung biopsies and absent from all non-COVID lung biopsies (open triangles) ranked from lowest to highest proportions of edits/reads, X-axis. Y axis is the proportion of edits to reads at each A-to-I editing site, P ​< ​0.0001, χ^2^ analysis. B) Number of unique A-to-I editing sites present in all COVID and all non-COVID lung biopsies, as in (A), P ​> ​0.05, χ^2^ analysis. C) Genome-wide A-to-I editing index of non-COVID and COVID lung biopsies. Editing index is the sum of all proportions of edits/reads in all samples, Open columns are unique editing sites, closed columns are shared editing sites, P ​< ​0.0001, unpaired *t*-test with Welch's correction.

We next analyzed A-to-I editing using RNA-seq data from lung biopsies from SARS-CoV-2 infected and uninfected patients. We identified >4000 A-to-I editing sites present in non-SARS-CoV-2 infected lung biopsies but only 63 A-to-I editing sites present in SARS-CoV-2 infected lung biopsies (Fig. 5A). We also identified 71 A-to-I editing sites shared between non-COVID-19 lung samples and COVID-19 lung samples (Fig. 5B). Of note, proportions of edits/reads at these shared sites were similar in the COVID-19 lung samples and the uninfected lung samples. We also applied the A-to-I editing index as a measure of total A-to-I editing across the entire genome to all samples. As above, the editing index was higher in the uninfected lung samples than the COVID-19 lung samples (Fig. 5C). Among the shared sites, the editing index was somewhat greater in the COVID-19 lung samples than the non-COVID-19 lung samples. Despite the limited number of samples, these results corroborate our findings in SARS-CoV-2 infected and mock-infected NHBE. Analyses of additional lung biopsies from COVID-19 patients with varying degrees of disease severity and from uninfected lung biopsies will be required to validate these findings.

## 4 Discussion

Loss of A-to-I editing of endogenous Alu RNAs and Alu RNA mediated activation of dsRNA sensors and subsequent downstream transcriptional responses is a potential source of inflammatory responses. Loss of A-to-I editing may occur in response to infections as well as in non-infectious diseases and syndromes such as autoimmune disease. Here, we report reduced levels of Alu RNA A-to-I editing resulting from SARS-CoV-2 infection in both NHBE culture models and in lung biopsies from COVID-19 patients compared to uninfected patients. Unedited endogenous Alu RNAs seen in SARS-CoV-2 infected NHBE are potent activators of inflammatory IRF and NF-kB transcriptional responses while these Alu RNAs, if edited, as seen in mock-infected NHBE, fail to activate these transcriptional responses. Thus, loss of A-to-I editing of Alu RNAs may represent a host pathway contributing to elevated inflammatory responses observed in patients with severe COVID-19 disease.

In general terms, viruses employ strategies to evade host recognition and activation of pathways that initiate anti-viral defenses (Garcia-Sastre, 2017). These initial anti-viral defenses culminate in induction of IFNs and ISGs to inhibit viral replication and other cytokines, chemokines and immune mediators to stimulate innate and adaptive arms of the immune system (Schoggins et al., 2011; tenOever, 2016). Strategies employed by SARS-CoV-2 to evade anti-viral responses include expression of viral proteins to inhibit mRNA splicing, mRNA translation and protein trafficking (Banerjee et al., 2020). These all appear to inhibit the IFN- response to viral infection. Interestingly, two proteins expressed by SARS-CoV-2, NSP8 and NSP9, bind to the 7SL RNA to inhibit protein trafficking. Since Alu RNAs arose from a head-to-tail fusion of the 7SL RNA and consist of two copies of the 7SL RNA, it seems possible that SARS-CoV-2 NSP8 and/or NSP9 may also bind Alu RNAs and this interaction may directly or indirectly interfere with ADAR catalyzed A-to-I editing of endogenous Alu RNAs.

Alternatively, other proteins produced from the SARS-CoV-2 genome may function to inhibit A-to-I editing either by direct inhibition of ADAR function or by other mechanisms. Numerous host RNA-binding proteins besides ADAR influence A-to-I editing (Quinones-Valdez et al., 2019). Examples of these genes and encoded proteins include *PUS1* (pseudouridine synthase 1), *FXR1* (fragile X mental retardation syndrome-related protein 1), *DROSHA* (drosha ribonuclease III), *TROVE2* (RO60), *EIF3D* (eukaryotic translation initiation factor 3 subunit D), *SAFB2* (scaffold attachment factor B2), *G3BP1* (G3BP stress granule assembly factor 1), and *PTBP1* (polypyrimidine tract binding protein 1). In general terms, certain RNA-binding proteins inhibit A-to-I editing at specific nucleotide sites (PUS1, G3BP1), while others stimulate A-to-I editing at specific nucleotide sites (FXR1, DROSHA, EI3D, PTBP1). A third group of RNA-binding proteins both stimulates and inhibits A-to-I editing depending upon the nucleotide site (TROVE2, SAFB2). This latter class somewhat resembles what is observed in SARS-CoV-2 infection of NHBE where editing at certain Alu RNAs is completely lost while editing at other Alu RNAs is unaffected and editing at other nucleotide sites is induced by SARS-CoV-2 infection.

These considerations also raise the question of whether or not regulation of A-to-I editing of Alu RNAs may represent a natural mechanism for the host to rapidly induce innate immune responses via activation of IRF- and NF-kB- transcriptional responses to combat both viral and bacterial infections (Li et al., 2016). In this model, high levels of Alu RNAs are continuously transcribed from the genome but are rapidly A-to-I edited to prevent activation of dsRNA sensors. Inhibition of A-to-I editing via endogenous mechanisms may result in rapid accumulation of unedited Alu RNAs, activation of dsRNA sensors, and induction of downstream inflammatory transcriptional responses. This notion is also supported by studies in multiple sclerosis that demonstrate decreased levels of A-to-I editing, increased levels of Alu RNAs that form double-stranded structures, and increased expression of IRF- and NF-kB regulated genes (Heinrich et al., 2019; Tossberg et al., 2020b). Of note, increased expression of IRF- and NF-kB regulated genes is a hallmark of many autoimmune diseases. Thus, partial loss of editing may represent a host response that activates innate immunity whereas greater loss of A-to-I editing may produce an innate immune response that is deleterious to the host leading to significant morbidity and even mortality as is seen in severe COVID-19 disease.

Alu:Alu IRs are major targets of A-to-I editing and we find that EELs with individual Alus and EELs with Alu:Alu IRs are both A-to-I edited in NHBE and editing of both classes of EELs is reduced in response to SARS-CoV-2 infection. Unedited Alu RNAs activate dsRNA sensors, RIG-I and TLR3 but minimally activate MDA5 while edited Alu RNAs fail to activate these dsRNA sensors (Tossberg, 2020b; Crooke, 2021). Alu:Alu IRs with perfect sequence homology also activate MDA5 (Ahmad, 2018). However, Alu:Alu inverted repeats with imperfect sequence homology fail to activate MDA5. Interestingly, one form of Aicardi-Goutières Syndrome caused by activating mutations in *IFIH1*, the gene that encodes MDA5, alters the function of MDA5 so mutant MDA5 now recognizes Alu:Alu inverted repeats with imperfect sequence homology. This results in continuous activation of IRF and NF-kB transcriptional paths, induction of IFNs, ISGs and other pro-inflammatory mediators and the severe symptoms associated with this syndrome.

Several limitations to this study exist and will need to be further explored. First, our analysis of lung biopsies is rather limited and would benefit from detailed analyses of bronchial lavages from patients with varying degrees of disease severity and viral load to determine associations between clinical parameters and levels of A-to-I editing. Second, correlations between levels of SARS-CoV-2 infection and levels of A-to-I editing, changes in levels of Alu RNA that form double-stranded structures, and IRF- and NF-kB induced transcriptional responses and induction of downstream target genes would lend further support to our model. Third, identification of either viral factors or host factors that mediate changes in A-to-I editing in response to SARS-CoV-2 infection has not been explored and represents an important next step that may offer the possibility of either stimulating or inhibiting A-to-I editing as a means to regulate levels of innate immune responses.

## Disclosures

The authors have no financial conflicts of interest.

## CRediT authorship contribution statement

**Philip S. Crooke:** Methodology, Formal analysis, Investigation, Writing – review & editing. **John T. Tossberg:** Methodology, Formal analysis, Investigation, Writing – review & editing, Supervision. **Krislyn P. Porter:** Formal analysis, Investigation, Writing – original draft, Writing – review & editing. **Thomas M. Aune:** Conceptualization, Methodology, Formal analysis, Writing – original draft, Writing – review & editing, Supervision, Project administration, Funding acquisition.

## Declaration of competing interest

The authors declare that they have no known competing financial interests or personal relationships that could have appeared to influence the work reported in this paper.
